# HIV-Related Oral Mucosa Lesions: A Cross-Sectional Study on a Cohort of Italian Patients

**DOI:** 10.3390/biomedicines12020436

**Published:** 2024-02-15

**Authors:** Marco Tarozzi, Elisa Baruzzi, Sem Decani, Camilla Tincati, Andrea Santoro, Laura Moneghini, Giovanni Lodi, Andrea Sardella, Antonio Carrassi, Elena Maria Varoni

**Affiliations:** 1 Dipartimento di Scienze Biomediche, Chirurgiche ed Odontoiatriche, Università degli Studi di Milano, 20142 Milano, Italy; marco.tarozzi@unimi.it (M.T.); elisa.baruzzi@unimi.it (E.B.); sem.decani@unimi.it (S.D.); giovanni.lodi@unimi.it (G.L.); andrea.sardella@unimi.it (A.S.); antonio.carrassi@unimi.it (A.C.); 2 Odontostomatologia II, ASST Santi Paolo e Carlo—Presidio Ospedaliero San Paolo, 20142 Milano, Italy; 3 Dipartimento di Scienze della Salute, Università degli Studi di Milano, 20122 Milano, Italy; camilla.tincati@unimi.it (C.T.); andrea.santoro1@unimi.it (A.S.); 4 Clinica di Malattie Infettive, ASST Santi Paolo e Carlo—Presidio Ospedaliero San Paolo, 20142 Milano, Italy; 5 Anatomia Patologica, ASST Santi Paolo e Carlo—Presidio Ospedaliero San Paolo, 20142 Milano, Italy; laura.moneghini@asst-santipaolocarlo.it

**Keywords:** HIV/AIDS, HPV, antiretroviral treatment, co-infection, oral medicine, oral diseases

## Abstract

Background: Human immunodeficiency virus (HIV) infection can be associated with oral mucosal diseases, including oral candidiasis and HPV infection, which are putative indicators of the immune status. Aim and Methods: This retrospective cross-sectional study was aimed at assessing the prevalence of HIV-related oral mucosal lesions in a cohort of Italian HIV+ patients regularly attending the Clinics of Infectious Diseases. Results: One hundred seventy-seven (n = 177) patients were enrolled and 30 (16.9%) of them showed HIV-related diseases of the oral mucosa. They were mainly found in male patients over 35 years old, undergoing Combination Antiretroviral Therapy (cART), and with CD4+ count < 500/µL. Oral candidiasis was the most common HIV-related oral lesion. No significant correlations could be detected between the prevalence of HPV infection and other clinical parameters (lymphocyte count, cART treatment and viral load). Conclusions: HIV-related oral mucosal diseases can correlate with immunosuppression. Early diagnosis and management of oral lesions in HIV+ patients should be part of the regular follow-up, from a multidisciplinary perspective of collaboration between oral medicine and infectious disease specialists, in an attempt to reduce morbidity due to oral lesions and modulate antiretroviral therapy according to the patient’s immune status.

## 1. Introduction

Acquired immunodeficiency syndrome (AIDS) is the symptomatic phase of the infection of human immunodeficiency virus (HIV), an RNA virus that impairs the patient’s immune system. HIV still represents a worldwide pandemic and AIDS is currently a major global health problem, having claimed about 630,000 lives and affecting 39 million people in 2022 [1]. The first case of AIDS was diagnosed in 1981, when young homosexual men started to die from uncommon opportunistic infections and rare tumours [2]. The disease was caused by HIV-1, while HIV-2 was identified as being responsible for a similar, less aggressive disease limited mostly to West Africa and strictly connected to a simian virus that causes immunodeficiency in captive macaques. AIDS is mainly a sexually transmitted disease. The constant and increasing HIV replication produces the virus- and immune-mediated destruction of the key immune effector cell, i.e., the CD4 lymphocyte. From a pathogenic point of view, indeed, the first event of HIV infection towards the target cell is represented by the binding of the viral surface glycoprotein (gp120) with the surface antigen cluster of differentiation 4 (CD4+), expressed mainly by the T-helper lymphocyte subpopulation [3]. Three stages of HIV infection have been recognized [2]. Acute HIV infection (symptomatic primary HIV or seroconversion) represents the earliest clinical manifestation of HIV infection, usually occurring in 2–4 weeks with symptoms similar to influenza or infectious mononucleosis and with high blood levels of HIV that correlate with an increased risk of transmission. The second stage is chronic HIV infection, also called asymptomatic HIV infection or clinical latency, since the virus multiplies at lower levels. Infected patients could be fully asymptomatic or manifest persistent generalized lymphadenopathy. Few individuals (below 0.5%) are “long-term non-progressors” with a stable CD4+ count and low levels of virus for several years. While patients taking antiretroviral therapy (ART) remain in the chronic stage for several decades, without ART, chronic HIV infection typically progresses to AIDS in a decade, along with the progressive decline in CD4+ count. The late phase of HIV infection is, thus, characterized by severe immunodeficiency, especially related to a decreased count of CD4+ cells, which act as a main marker of disease progression [4]. According to disease stages, the CD4+ cell count decreases by about 30%, whereas the CD8+ (cluster of differentiation 8) cell count increases by about 40%, resulting in an inverted CD4+/CD8+ cell ratio that is usually less than 1. The normal CD4+/CD8+ ratio in healthy individuals can range between 1.5 and 2.5, with a wide heterogeneity related to sex, age, ethnicity, genetics, exposures, and infections [5]. During HIV infection, the immune system is, thus, depleted, leaving the patient vulnerable to opportunistic infections [6]. At 350 cells/µL, the susceptibility to pathogens, such as Mycobacterium tuberculosis, oral and vaginal candidiasis and varicella zoster virus, increases; at CD4+ count < 200 cells/µL, the individual can develop life-threatening infections (*Pneumocystis jirovecii* pneumonia or cerebral toxoplasmosis) or HIV-related malignancies (Kaposi’s sarcoma) [2].

The oral mucosal manifestations of HIV in particular can be, for the patient, the first sign of the disease, although they are associated with AIDS. Therefore, the importance of recognizing HIV-related oral lesions is pivotal for the diagnosis of the underlying disease, as it also occurs in other infections or chronic disorders for which oral lesions may represent an important clinical manifestation: COVID-19, monkeypox, scarlet fever, syphilis, and inflammatory bowel diseases including Crohn’s disease and ulcerative colitis [7,8,9,10,11,12]. Nonetheless, the detection of HIV-related oral lesions can also be useful in evaluating disease progression and, representing the effects of a reduced immune competence, could play an important role as indicators of the effectiveness of antiretroviral therapy [13,14,15]. 

With the advent of new pharmacological therapies against HIV, a strong decline in the frequency and in the severity of the oral manifestations typical of HIV infection has been observed. Studies from both the European and American continents report a reduction in the frequency of HIV-related oral manifestations ranging between 10 and 50% [16,17]. A large cohort study was conducted on 1000 patients in Mexico City; the authors observed the effects on the oral mucosa of Combination Antiretroviral Therapy (cART) over a period of 12 years, showing a reduction in the prevalence of oral candidiasis by 50% [17]. The positive effect of cART therapy on oral cavity lesions has been analysed in several prospective and retrospective studies, showing a correlation with the severity of the lesions [18]. cART, in particular, plays a fundamental role in the control of oral candidiasis; a study conducted in 2010 on 142 naïve Nigerian individuals receiving cART showed a prevalence of oral candidiasis of 22.4% before starting the antiretroviral therapy and the resolution of all cases by the third month after the beginning of the therapy [19]. Antiretroviral therapies have a marked effect against candidiasis, reducing both the incidence and the recurrence [18,20,21,22,23]. Also hairy leucoplakia showed a decline in prevalence, although this condition demonstrated a slower response to cART: a gradual reduction in the size of the lesions was observed during the first months of therapy up to the complete remission achieved at the end of the fifth month [19]. Similarly, Kaposi’s sarcoma was associated with a reduction in its incidence, from 9% to 1%, after cART, and analogous decreasing trends have been reported for salivary gland diseases and periodontal disease [16,19]. On the other hand, melanotic hyperpigmentation is found more frequently in individuals receiving cART, probably due to an overstimulation of the hormone responsible for the production of melanin (melanocyte-stimulating hormone, MSH) [19,20,24]. Further oral manifestations associated with HIV are HPV-related lesions [18]. A cross-sectional study performed in Mexico described 55 cases out 787 (6.9%) of HPV-related oral lesions, which were independently associated with age (≥40 years) and with a longer time of cART (≥12 months) [25]. Cross-sectional studies on Italian HIV+ men (predominantly men having sex with men, MSM) showed that oral HPV DNA could be detected in approximately 20% of cases, which is to a much lower extent compared to anal sites [26,27,28,29]. To interpret this data, it is important to highlight that also the global prevalence of oral HPV infection in healthy individuals can be relevant, ranging from 0.67% to 35% [30]; the oral cavity contains a wide spectrum of HPVs, with beta types representing the predominant genus [31]. In the general population, HPV has been found to be more prevalent in males, while subclinical oral HPV infection was detected more frequently in adults aged under 40 or over 60 years [32].

However, data are still scanty on the prevalence of the different oral mucosa lesions in HIV+ Italian patients; previous studies were published in the early 2000s [33,34,35]. In this context, the aim of our retrospective study is to provide most recent data on the prevalence of HIV-related oral lesions in an Italian cohort of HIV patients, who regularly attended the infectious disease hospital clinics.

## 2. Materials and Methods

This retrospective cross-sectional study was conducted by consulting a clinical database of HIV+ patients, who were referred to the clinical unit of infectious diseases at the San Paolo Hospital (Milan, Italy) and who also attended dental visits at the dental clinics in the same hospital (from 2008 to 2012). This brief report is a spin-off of a previous study on HPV mucosal infection at genital and oral sites of an Italian cohort of HIV+ patients [28].

### 2.1. Data Collection

Each patient’s medical record contained socio-demographic data, medical history (other co-morbidities and pharmacological therapies, including cART details), route of HIV infection, clinical details related to HIV infection (date of diagnosis, CD4+ count and viremia of the past 4 months, AIDS clinical stage) and clinical details related to oral examinations (i.e., presence of oral mucosa lesions). Specific information on the HIV pharmacological therapy, in particular, was recorded for each patient as well as the duration of HIV+ (HIV duration, in months). The cytopathological findings of the oral scraping, performed to detect oral HPV, were also recorded as well as the histopathological diagnosis of oral mucosal lesions, when required. The following HIV-related oral mucosal lesions were considered: any form of oral candidiasis (including erythematous, pseudomembranous and atrophic forms, angular cheilitis, median rhomboid glossitis and prosthetic stomatitis), hairy leucoplakia, oral squamous papilloma, herpetic lesions and further lesions associable to cART treatment, including lichenoid lesions and mucosal hyperpigmentation. Other oral mucosal lesions were not considered attributable to HIV health status or therapy and were classified as “not HIV-related”, e.g., geographic tongue, aphthous ulcers, traumatic ulcers, haemangiomas, traumatic fibromas, frictional hyperkeratosis, amalgam tattoo and oral leucoplakia (not hairy).

### 2.2. Clinical Oral Examination, Specimen Collection and Cyto/Histopathological Analyses

Two well-trained and calibrated oral medicine specialists, using a dental examination kit (mirror, probe and tweezers) and sterile gauze, performed the clinical examination of the oral mucosa. The clinical diagnosis was achieved in agreement between the two. The oral scraping for HPV virology analysis was performed using a disposable dermatological spatula, which is a scalpel with a ring blade of 7 mm in diameter. The specimen was dipped in the Preserve-Cyt^®^ (Marlborough, USA) fixative liquid and sent to the pathology unit for the detection of HPV in the oral cavity. In case of HPV detection, HPV genotyping was also performed to assess the presence of high-risk HPV types (18, 31, 33, 35, 52, 53, 56, 58, 66, 67, 68, 73, 83) versus low-risk HPV types (6, 11, 54, 56, 61, 62, 71, 72, 74, 80, 81, 82, 84, 85, 86, 89, 107, SIBX1, SIBX3). In presence of oral mucosal lesions, a diagnostic biopsy was performed, when required, and sent to the histopathology unit in order to confirm the clinical hypothesis.

### 2.3. Statistical Analysis

A Fisher’s test was performed to analyse the possible association between the lymphocyte count and cART treatment with the presence of HIV-related oral lesions, while the Student’s *t*-test was used to compare the average viral load of HPV-infected and non-HPV-infected individuals with the presence/absence of oral lesions. A Chi-square test was applied to compare percentages. Statistical significance was set at *p* < 0.05.

## 3. Results

We enrolled 177 patients, of whom 132 (74.6%) were males and 45 (25.4%) females (age ranging from 21 to 69 years); the mean HIV+ duration was 107 ± 90 months. One hundred (56.5%) of the patients were homosexual, while 77 (43.5%) were heterosexual. One hundred forty (n = 140; 79%) were taking cART, while 37 (21%) were naive to the drug treatment. The main route of transmission was related to sexual behaviour, with men having sex with men (MSM) being the most represented group. Socio-demographic data and clinical HIV status are reported in Table 1. Patients had a mean viral load of 26,186 ± 767 copies of HIV-RNA/mL (range: 704,797–39 copies of HIV-RNA/mL).

### 3.1. Oral HPV Infection

HPV prevalence was slightly higher in men (21%) than in women (15.5%) (*p* = 0.4); no significant difference could be found between homosexual and heterosexual individuals. The LR genotypes were found to be slightly prevalent in men, representing 57% of the total infections, while the HR genotypes were slightly more represented in women (57%), although without a statistically significant difference between sexes (*p* = 0.4). The age group between 36 and 42 years showed the highest prevalence of LR–HPV infections (70%, n = 7), while HR–HPVs were slightly more prevalent in patients over the age of 51 years (55.5%, n = 5). Comparing the group taking cART therapy and the group not under antiretroviral drugs, the prevalence of HPV infection was 21.4% in the former and 13.5% in the latter; the association between cART treatment and the prevalence of HPV infection was not statistically significant (*p* = 0.5). Considering the prevalence of oral HPV infection in individuals with a lymphocyte count less than 500 cells/µL or higher than 500 cells/µL, 23.5% of patients with a high lymphocyte count showed HPV infection, while among people with a lymphocyte count below 500 CD4+/µL the prevalence was 16.3%. This result was not statistically significant (*p* = 0.2).

Table 2 summarizes the distribution of high-risk HPV serotypes and low-risk HPV in the population.

Patients diagnosed with oral HPV infection had a mean HIV viral load of 43,641.4 HIV RNA copies (range: 39–631,249 HIV RNA copies). The comparison of the viral load between HPV+ and HPV− patients was not statistically significant (*p* = 0.2). 

### 3.2. Oral Mucosal Lesions

**Figure 1:**
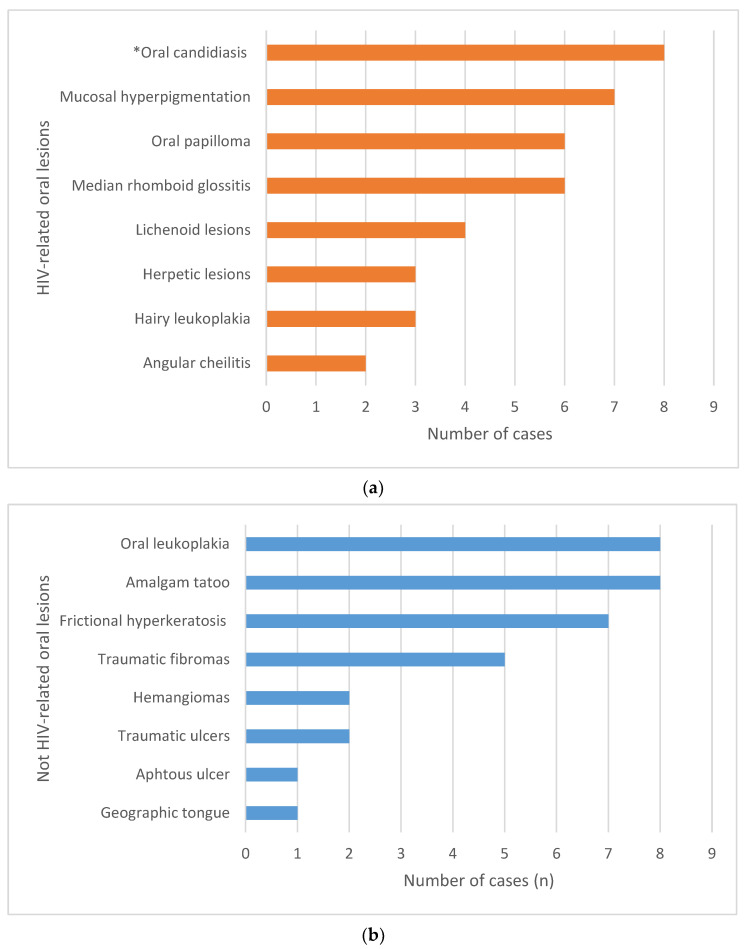
Frequency of oral mucosa lesions in the cohort of patients enrolled in the study: (**a**) HIV-related oral mucosa lesions (* the group includes erythematous and/or pseudomembranous candidiasis); (**b**) non-HIV-related oral mucosa lesions.

Fifty-six (n = 56) patients out of 177 (31.6%) showed oral mucosa lesions; in 21 cases, the patient had more than one lesion. Of these 56 patients, thirty (53.6%) showed HIV-related oral mucosa lesions (Figure 1a), while the remaining twenty-six (46.4%) showed oral lesions not directly associated with HIV (Figure 1b). The overall prevalence of HIV-related oral mucosa lesions in this cohort of HIV+ patients was 16.9% (30/177). Among HIV-associated oral lesions, the most prevalent ones were oral candidiasis (n = 8), oral hyperpigmentation (n = 7), rhomboid median glossitis (n = 6) and oral papilloma (n = 6). 

One patient reported a diagnosis of Burkitt’s lymphoma a decade earlier.

Table 3 describes the demographic and clinical characteristics of patients with at least one oral mucosal lesion associated with HIV. Most of them were males, over 35 years old, undergoing cART and with a CD4+ count < 500/µL.

## 4. Discussion

This cross-sectional study showed, in an Italian group of HIV+ individuals, the prevalence of HIV-related oral mucosal lesions. The prevalence of oral HPV co-infection was also reported.

HIV-related oral mucosal lesions were identified in 30 out of 177 patients (16.9%), a figure that is lower than those reported in previous studies, reaching a prevalence up to 85% [24,33,35,36,37,38,39]. This difference can be ascribed to the improvements in cART therapy, which has showed beneficial effects in reducing the oral mucosal lesions [18], and the general compliance of the patients. Further, the present study was conducted in a period during which literature evidence suggested that cART introduction in asymptomatic patients with high CD4+ T-cell counts had clinical and viro-immunological benefits; findings later corroborated in the START trial [40], thus leading clinicians to introduce cART earlier in the course of disease. Overall, in our cohort, most of patients presenting HIV-related oral mucosa lesions were males with a lymphocyte count below 500 cells/µL. This is consistent with previous studies supporting that the prevalence of HIV-related lesions and their severity can correlate with the patient’s immunodeficiency [13,15,37,39,41]. 

Oral candidiasis was one of the most prevalent oral lesions in our cohort of patients (8 cases out 177; 4.5%); however, our study showed a lower prevalence compared to previous studies [24,36,37,38,39,42]. Oral mucosa hyperpigmentation (7/177; 3.9%), as an adverse drug reaction to cART, represented another common finding, again at a lower percentage than previous epidemiological data [22,24,31,43,44]. Oral hairy leucoplakia was found in 1.7% of patients, within the range available in literature, varying from 0.5% to 11.5% [24,38,39,45]. Since hairy leucoplakia is closely associated with severe immunodeficiency, the low prevalence in our study can be, in particular, ascribed to the careful monitoring and pharmacological management of these patients.

Oral papilloma was detected in 6 of the 177 patients (6/177; 3.4%), with a similar rate to previous works, reporting a frequency of wart-like lesions around 3–6% [24,33,35,46]. Regarding the prevalence of HPV infection in HIV+ patients, our study showed a prevalence of 19.7%, in line with values reported in literature, ranging from 11% to 35% [26,47,48,49,50,51]. As expected, the prevalence was higher than that reported, on average, for the general population (4.8%) [48], although literature shows that HPV infection can be prevalent also in healthy individuals, with highly variable percentages [30]. The difference between general and HIV+ populations, however, can be explained by the higher virulence of HPV in HIV+ patients, which is due to a reduced host immune response, an increased susceptibility to infection, and to the direct interactions between the two viruses, e.g., HIV is able to increase the expression of HPV E6 and E7 oncogenes [52]. Analysing HPV viral genotypes having different oncogenic potential, the literature described a prevalence of infection with HR–HPV genotypes in HIV-positive individuals that was between 13.7% and 36.8% [29,47,49,51], while for LR–HPV genotypes the infection rate ranged from 26.3% to 66.6% [29]. Our cohort of patients showed lower prevalence rates for both HR– and LR–HPV, at 9% and 10.7%, respectively. The most frequently detected oral HPV types were HPV66 and HPV33 among HR, and HPV32, HPV72, HPV11 and HPV61 among LR. HPV16 was found in only 1% of cases. This finding differed from previous studies that showed HPV16 as the predominant type [6,33] and may have epidemiological implications in term of cancer risk. HPV16 has been detected in more than 85% of HPV-related OSSC [6], and a case–control study estimated that oral HPV16 correlates with a 50-fold increase in the odds of HPV-driven oro-pharyngeal cancer [31].

The current study described the types and the frequency of oral mucosal diseases in a large cohort of HIV+ patients, as assessed by oral medicine specialists working in close collaboration with infectious disease specialists. Oral findings were matched with clinical parameters related to HIV in order to corroborate the utility of oral mucosa screening in the early detection of oral lesions as a manifestation of patient’s immunodeficiency. Study limitations include the absence of a control group to compare the prevalence of oral diseases and HPV infection, the retrospective design and the difficulty in the generalization of findings, since this study referred to a single Italian clinical centre, recruiting patients from 2008 to 2012.

Oral mucosal diseases might indicate the occurrence of underlying systemic diseases, of which often they represent the first pathological sign or the exacerbation. The importance of early detection is pivotal for a prompt diagnosis and for implementing the treatment strategy. Besides HIV, other infectious diseases can be associated with the occurrence of oral lesions, such as oral ulcers in the case of COVID-19 and monkey pox [7,8], or the so-called “strawberry tongue” as a pathognomonic finding of scarlet fever [9]. Again, inflammatory bowel diseases, such as Crohn’s disease and ulcerative colitis, are also associated with the presence of recurrent aphthous stomatitis [11,12]. A complete intraoral examination, including dental, periodontal and oral mucosa assessment, is always recommended to detect any signs that are suggestive of a systemic condition. Nonetheless, the correct management of each individual should include a multidisciplinary approach, with close collaboration between oral medicine and infectious disease specialists, in order to treat the patient globally. Emphasis should be also given to the importance of maintaining a high standard of oral health [53,54] with periodical dental check-ups.

## 5. Conclusions

This study describes the frequency of oral mucosa diseases in an Italian cohort of HIV+ patients. Among HIV-associated oral lesions, the most prevalent one was oral candidiasis. The patients with HIV-related oral lesions were mainly males, over 35 years old, undergoing cART and with a CD4+ count < 500/µL. No statistically significant associations could be found between the prevalence of HPV infection and HIV-related clinical parameters (lymphocyte count, cART treatment and viral load).

## Figures and Tables

**Table 1:** Characteristics of the study population (n = 177): gender, age groups, whether or not they are undergoing antiretroviral therapy, and CD4+ count.

Demographic Characteristics	n (%)
**Sex**	
Males	132 (74.6%)
Females	45 (25.4%)
**Age**	
Under 35	43 (24.3%)
35–42	48 (27.1%)
43–50	52 (29.4%)
Over 51	34 (19.2%)
**cART**	
Yes	140 (79%)
No	37 (21%)
**CD4+ (cells/µL)**	
Under 200	12 (6.8%)
200–500	80 (45.2%)
Over 500	85 (48%)
Route of transmission *	
MSM	100 (56.5%)
MSW or WSM	52 (29.38%)
IDUs	23 (13%)
Vertical trasmission	1 (0.56%)
Transfusion	1 (0.56%)

* Men having sex with men (MSM); men having sex with women (MSW); women having sex with men (WSM); intravenous drug users (IDUs).

**Table 2:** Prevalence of HPV in the study population (n = 177).

Demographic Characteristics	n			
	HPV−	HPV+	LR-HPV	HR-HPV
**Sex**				
Males	104	28	16	12
Females	38	7	3	4
**Age**				
Under 35	35	8	4	4
36–42	38	10	7	3
43–50	44	8	4	4
Over 51	25	9	4	5
**cART**				
Yes	110	30	16	14
No	32	5	3	2
**CD4+ (cells/µL)**				
Under 200	11	1	0	1
200–500	66	14	4	10
Over 500	65	20	12	8
**Total**	142	35	19	16

**Table 3:** Demographic characteristics of patients with HIV-related oral mucosal lesions (n = 30).

Demographic and Clinical Characteristics	Number of Patients (%)
**Sex**	
Males	22 (73.3%)
Females	8 (26.7%)
**Age**	
Under 35	5 (16.7%)
35–42	2 (6.6)
43–50	8 (26.7%)
Over 51	15 (50%)
**cART**	
Yes	26 (86.7%)
No	4 (13.3%)
**CD4+ (cells/µL)**	
Under 200	4 (13.3%)
200–500	14 (46.7%)
Over 500	12 (40%)

## Data Availability

Data are available on request due to restrictions, e.g., privacy or ethical.
